# Integrative analysis of morphological, transcriptomic, and metabolomic approaches to uncover the function of flavonoids in the salt stress response of *Alhagi camelorum*

**DOI:** 10.3389/fpls.2025.1678456

**Published:** 2026-01-05

**Authors:** Gangliang Tang, Lanlan Long, Xiangyi Li, Xiaoxue Guo, Mengxiao Lu, Fanjiang Zeng, Noor Muhammad, Bo Zhang

**Affiliations:** 1 State Key Laboratory of Desert and Oasis Ecology, Key Laboratory of Ecological Safety and Sustainable Development in Arid Lands, Xinjiang Institute of Ecology and Geography, Chinese Academy of Sciences, Urumqi, China; 2 Xinjiang Key Laboratory of Desert Plant Roots Ecology and Vegetation Restoration, Xinjiang Institute of Ecology and Geography, Chinese Academy of Sciences, Urumqi, China; 3 Cele National Station of Observation and Research for Desert-Grassland Ecosystems, Cele, China; 4 College of Horticulture, Hebei Agricultural University, Baoding, Hebei, China; 5 College of Forestry, Hebei Agricultural University, Baoding, Hebei, China; 6 National Engineering Technology Research Center for Desert-Oasis Ecological Construction, Xinjiang Institute of Ecology and Geography, Chinese Academy of Sciences, Urumqi, China

**Keywords:** *Alhagi camelorum*, salt stress, gene expression, qPCR, flavonoid

## Abstract

Salinity or salt stress significantly influences plant productivity, growth, and development, including that of *Alhagi camelorum*. In the current study, transcriptomic characterization discovered DEGs among the samples of four paired groups. During the metabolomic profiling, the top ten identified primary metabolites are amino acids and their derivatives, fatty acids, alcohols and amines, lipids, GP, heterocyclic compounds, and flavonoids. An analysis of comparative metabolic pathways and KEGG enrichment indicated that salt stress disrupts the biosynthesis of sesquiterpenoids, triterpenoids, and diterpenoids, as well as the metabolism of phenylalanine, etc. Notably, the pathways associated with flavonoid biosynthesis, including genes such as (*Asp04G007070, Asp04G008880, Asp06G017850, Asp02G032990, Asp05G012840, Asp03G022990, Asp05G008310, Asp05G006550*) were identified as the most significant key genes. These findings underscore the molecular mechanisms involved in the salt response of *A. camelorum*, which could be employed in conservation programs for *A. camelorum* to enhance its tolerance to saline conditions.

## 1 Introduction

The species of genus *Alhagi*, known as camelthorn, are perennial shrubs that thrive in arid environments, especially in the desert (Ullah et al., 2022). It belongs to the Fabaceae family, and the small flowers with pinkish peduncles and a profusion of thorns set it apart (Tang et al., 2024). The top portions of the plants are where the blooms are found (Muhammad et al., 2015; Tariq et al., 2022). The camelthorn’s fruits can be either brown or scarlet in color; the seed reproduces quickly and is extensively dispersed (Tang et al., 2024; Tariq et al., 2022). In addition, the perennial camelthorn has a wide geographic distribution as it stops soil erosion which is crucial for protecting sandy slopes. Moreover, the herb is well known for its medicinal properties and is frequently used to treat kidney stones, rheumatism, and gastrointestinal disorders (Asghari et al., 2016). *A. camelorum* Fisch., a leguminous species, is a multi-branched semi-shrub typically found in arid regions of Central Asia, especially in northwest China, northern India, Kazakhstan, Afghanistan, Iran, Pakistan, Iraq, Mongolia, Syria, and beyond (Manafu et al., 2024; Srivastava et al., 2014). In China, *A. camelorum* is primarily distributed in the Shanshan and Aksu areas of Xinjiang (Manafu et al., 2024; Srivastava et al., 2014). In recent years, it has been determined that *A. camelorum*, one of the six major species of camelthorn, is abundant in bioactive substances such as flavonoids, alkaloids, saponins, and tannins (Pirasteh-Anosheh et al., 2022; Tang et al., 2024). These substances support its antibacterial, anti-inflammatory, antioxidant qualities and response to various stresses (Tang et al., 2024; Ullah et al., 2022).

Stress factors—such as drought, extreme temperatures, salinity, pests, and physical damage—can significantly impact plant growth and development (Wang et al., 2024a).The accumulation of water-soluble salts (Wang et al., 2023), such as sodium (Na^+^), potassium (K^+^), chloride (Cl^−^), and sulfate (SO_4_^2−^), in the root zone is the primary cause of soil salinization (Munns and Tester, 2008). Osmotic changes brought on by this accumulation make it more difficult for plant root cells to absorb water from the soil (Kamran et al., 2019; Munns and Tester, 2008; Stavi et al., 2021). The presence of salt ions causes hyperionic salt stress, which can be detrimental to plant cells. Na^+^ and Cl^−^ are the key water-soluble salts that contribute to soil salinity. Notably, elevated levels of Na^+^ among the exchangeable cations lead to sodicity (Balasubramaniam et al., 2023). While naturally occurring salt-tolerant plants, known as halophytes, may flourish in extremely salty settings, many terrestrial plants can withstand low to moderate salinity levels. The majority of contemporary crop species, on the other hand, are categorized as glycophytes and struggle to thrive in salinized environments. Many cultivated crops are especially susceptible to soil salinity, including rice and tomatoes. Short-term osmotic stress is the first of salinity’s harmful effects on plants, which are followed over time by the slow accumulation of poisonous ions (Andrade Foronda, 2022; Ullah et al., 2021).

Osmotic stress occurs in the early phases of salt exposure as a result of the slow absorption of salts, which reduces the root zone’s water potential (Abbasi et al., 2016; Ullah et al., 2021). Stunted growth is the primary consequence of this decrease in water potential, as it leads to a reduced ability of plant cells to absorb water. Long-term excessive salinity damages plant cells and tissues by causing a buildup of poisonous ions, including Na^+^, Cl^−^, and SO_4_^2−^ (Isayenkov and Maathuis, 2019). These ions can induce ion toxicity and disrupt nutrient absorption (Ji et al., 2022). Salt stress has detrimental effects on plants in a number of ways, including morphologically (stunted growth, chlorosis, and poor seed germination); physiologically (photosynthesis is suppressed and nutrients are out of balance); and biochemically (oxidative stress, electrolyte leakage, and membrane disruption) (Hannachi et al., 2022; Ji et al., 2022).

Salt stress is a significant limiting factor that restricts the distribution and growth of many plants. Moreover, salt stress is a significant limiting factor that restricts the distribution and growth of many plants. However, for *A. camelorum* plants, due to their high drought tolerance with long main root distribution, the salt response of A. camelorum has not been systematically studied. Understanding the salt stress response is a key focus in the genetic and breeding of *A. camelorum*. So far, both traditional breeding methods and modern genetic engineering techniques have made considerable progress in developing plants that are more resilient to salt. Studying salt tolerance in *A. camelorum* is crucial as it enhances our understanding of the intricate mechanisms that facilitate salt tolerance in this plant species. This research can also lead to the development of strategies aimed at reducing the adverse effects of salt stress on *A. camelorum*. Therefore, this study hypothesizes that salt stress induces specific physiological, transcriptomic, and metabolomic changes in *A.camelorum* that underlie its adaptive mechanisms to saline conditions. Specifically, we aim to identify differentially expressed genes and key metabolic pathways, such as flavonoid biosynthesis and terpenoid metabolism, that contribute to salt tolerance. These insights may potentially facilitate future efforts to develop highly salt-tolerant *A. camelorum* plants.

## 2 Materials and methods

### 2.1 Plant material and salt treatment

The seeds of *A. camelorum* were obtained at Cele National Station of Observation and Research for Desert-grassland Ecosystems, Cele, Xinjiang, China. The seeds were soaked in sterile water for 30 seconds, then sterilized with 0.1% mercuric chloride for 10 minutes and rinsed 3 times with sterile water. Further, the seeds were sterilized with 75% alcohol for 30 seconds and rinsed three times with sterile water. The seeds were planted on MS medium, which contained MS + 20.0g/L sucrose+ 5.5 g/L agar (pH=5.8) under a 16-h photoperiod, temperature of 25 °C. After twenty-one days of cultivation, the plants were treated with 100 and 200 mM NaCl, respectively. The whole tissue samples of 0, 2, 4, and 6 days were collected for morphological, transcriptome, and metabolome analysis; meanwhile, the untreated (water) samples were taken as a negative control.

### 2.2 Morphological analysis

The phenotype of *A. camelorum* and its responding parameters, including the number of leaves, the length of shoots, the length of main roots, and the number of lateral roots, in response to 100 mM and 200 mM NaCl treatments on the sixth day, were measured, and the statistical analysis was performed (Tang et al., 2024).

### 2.3 Transcriptomic characterization

Total RNA was isolated from the *A. camelorum* seedlings with controls and 200 mM NaCl-treated triplicate samples following the methodology of Tang et al. (2024). The main methods for RNA quality control were quantitative analysis using Nanodrop and detection of RNA integrity using Agilent 4200 TapeStation.

### 2.4 Transcriptomic data analysis

The samples of 0, 2, 4, and 6 days under 200 mM NaCl treatment were selected to do transcriptomic analysis, which were designated as NaCl_1, NaCl_2, NaCl_3 and NaCl_4, respectively. The untreated (water) samples of 0, 2, 4, and 6 days were designated as Con_1, Con_2, Con_3 and Con_4, respectively. The transcriptome analysis was performed by Chi Biotech Company, Guangzhou, China (Wang et al., 2025). Briefly, after purification, the double-stranded cDNA was subjected to terminal repair, followed by the addition of an A tail and the connection of sequencing adapters. cDNA of around 200–300 bp was screened and amplified by PCR, and the PCR product was purified again to obtain a library. Following the completion of library construction, the Qubit 2.0 Fluorometer was used for quantification, and the library was diluted to 1.5 ng/μL. The Agilent 4200 TapeStation was then employed to assess the insert size of the library to ensure its quality. Once the library passed the quality check, Illumina sequencing was conducted on the samples using the PE150 sequencing mode.

The raw reads were first processed using the cutadapt software v4.3 to eliminate adapters and low-quality sequences. The resulting clean reads were then aligned to the reference genome of *A. camelorum* using the STAR aligner v2.7.10b (Dobin et al., 2013). The RSEM program was utilized to assess gene transcription levels as measured by transcripts per kilobase of exon model per million mapped reads (TPM). PCA analysis used the linear algebra calculation method to perform dimensionality reduction and principal component extraction on tens of thousands of gene variables. For differential expression analysis of genes (DEGs) between two sample groups, the edgeR software was employed, applying a corrected *P*-value threshold of 0.05 with |log2(FoldChange)| > 1. Venn diagrams were constructed using the R package VennDiagram v1.7.3. GO and KEGG enrichment analyses for the DEGs were performed using the Goatools and KOBAS software, respectively. The visualization of DEGs was conducted using TBtools (Chen et al., 2020).

### 2.5 Metabolomic analysis

The samples of 0 and 4 days under 200 mM NaCl treatment were selected to do metabolomic analysis, which were designated as NaCl_1 and NaCl_3, respectively. The untreated (water) samples of 0 and 4 days were designated as Con_1 and Con_3, respectively. Four replications for each sample were conducted for the metabolomic analysis by Chi Biotech Company, Guangzhou, China. Briefly, weighed 50 mg of sample powder and added 1200 μL 70% pre-cooled methanol-water internal standard extraction solution. After centrifugation (at a speed of 12000 rpm for 3 mins), the supernatant was collected and filtered using a microporous membrane (0.22 μm pore). Filtering the sample and storing it in an injection bottle for UPLC-MS/MS analysis at the following conditions: Chromatography Column (Waters ACQUITY Premier HSS T3 Column, 1.8 µm), Mobile phase (A: 0.1% formic acid/water; Mobile phase B: 0.1% formic acid/acetonitrile) and the instrument column temperature was 40 °C, the flow rate was 0.4 mL/min and the injection volume was 4 μL. The Variable Importance in the Projection (VIP) parameter was utilized to evaluate the relative significance of each metabolite within the OPLS-DA model. Metabolites were classified as differentially accumulated metabolites (DAMs) if they had a VIP value of ≥ 1 and a P-value of ≤ 0.05 (Wang P. et al., 2020).

### 2.6 qPCR analysis of flavonoid biosynthesis genes in response to salt stress

The qPCR analysis of flavonoid biosynthesis genes in response to salt stress was performed, which could refer to the method described by Wang et al. (2024b). The *Asp05G026640* was selected as a housekeeping gene, and the relative expression level was calculated by 2^-△△Ct^, and the primer sequence is shown in **Table 1**.

**Table 1:** The primer sequences used for qPCR analysis.

Gene id	Forward primers (5’-3’)	Reverse primers (5’-3’)
*Asp02G032990*	CAGCTACCGCTCTAGTGAAGG	GTTCACCCCACTTGTCAATGC
*Asp03G022990*	TATCTGCCGTCGGATCAAGTG	AGCAGGGCCTTCTCTGAAATC
*Asp04G007070*	CTGAGAACCATCCCCATCTCG	GGGAATGAAAACTTTCCGGGC
*Asp04G008880*	CTGCCGATGTTTCTTCTGCAC	TGGTCCAGACTGATTCTTGCC
*Asp05G006550*	GATGGCAAATGGCGAAGATCC	ACTCTATGATGCTGGCGGATG
*Asp05G008310*	AGCAAAAGTCAACCACGATGC	CTGCTTCCAACAACACTGCTC
*Asp05G012840*	TAACCGCCCTATTCGTAACCG	ACTTGGAGCCAGTTACCGAAG
*Asp06G017850*	TCAAGGTTGGTGGAGATGGTG	CACCACGAAGACACCAAAACC
*Asp05G026640*	GTGGTGGCTCAACTATGT	TCCTCCAATCCAGACACT

### 2.7 Statistical analysis

A one-way Student’s t-test was performed to assess the data of the morphological analysis using GraphPad Prism 8 software.

## 3 Results

### 3.1 Effect of salt stress on the growth morphological parameters of *A. camelorum* seedlings

**Figure 1:**
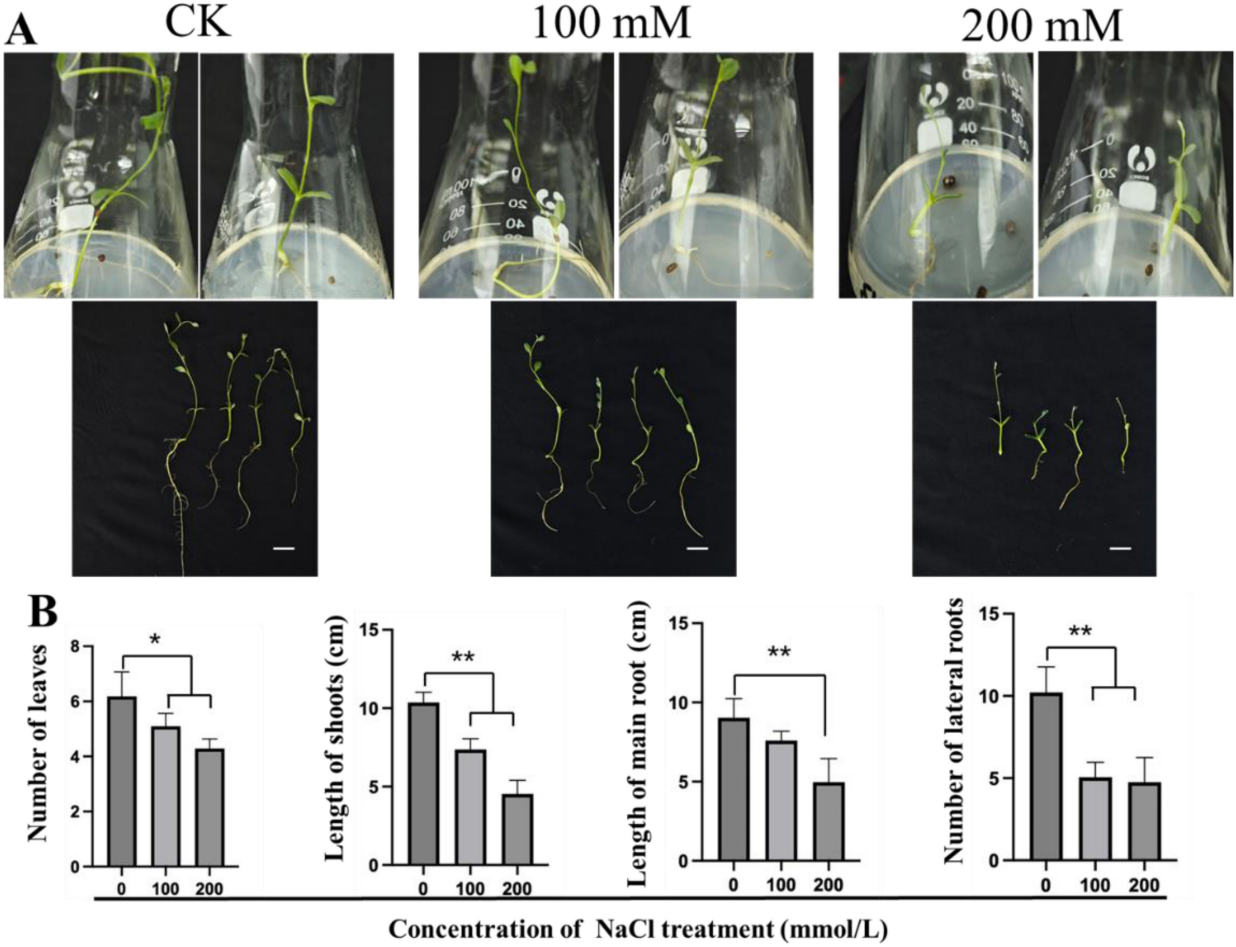
The morphological analysis of *Alhagi camelorum* in response to salt stress. **(A)**. The phenotype of *A. camelorum* under 100 mM and 200 mM NaCl treatments on the sixth day, respectively, and the untreated samples were taken as control (CK). **(B)**. The number of leaves, lengths of shoots, length of main root, and number of lateral roots under 100 mM and 200 mM NaCl treatments were observed on the sixth day, respectively. Data represent the mean value ± SE from three independent replications. * and ** indicates significant differences at *p* < 0.05 and *p* < 0.01 level, respectively.

To get insight into the effect of salt stress on the growth status of *A. camelorum*, the morphological parameters after 100 mM and 200 mM NaCl treatment after six days were analyzed. The growth of *A. camelorum* seedlings was notably hindered or inhibited under NaCl stress compared to the control group (**Figure 1A**). Specifically, the treatments with 100 mM and 200 mM NaCl resulted in a decrease in the number of leaves, shoot lengths, main root length, and the number of lateral roots after six days (**Figure 1B**), which indicated the salt stress had a significantly negative effect on the growth of shoots and roots of *A. camelorum*.

### 3.2 Transcriptome data quality assessment

As 200 mM NaCl treatment has a more significant effect on the growth of *A. camelorum*, four-time point samples were collected to do the transcriptome analysis to reveal the molecular mechanism under it. A total of twenty-four cDNA libraries were constructed from the *A. camelorum* samples, including both treatment and control groups, using total RNA isolated from these samples. As shown in **Supplementary Data Sheet 1**, the raw reads for each treatment exceeded 19400000, while the adapter-trimmed clean reads also surpassed 1.94×10^7^, resulting in a clean rate of over 99.97%. The mass fraction percentage of Q30 bases was greater than 95.04%, and the GC content ranged from 43.34% to 44.16%. Additionally, the matching efficiency between the clean reads of each sample and the reference gene ranged from 95.36% to 96.54%. These results indicated that the transcriptome sequencing data were of high quality and were suitable for subsequent analysis.

### 3.3 Correlation and PCA analysis among samples

**Figure 2:**
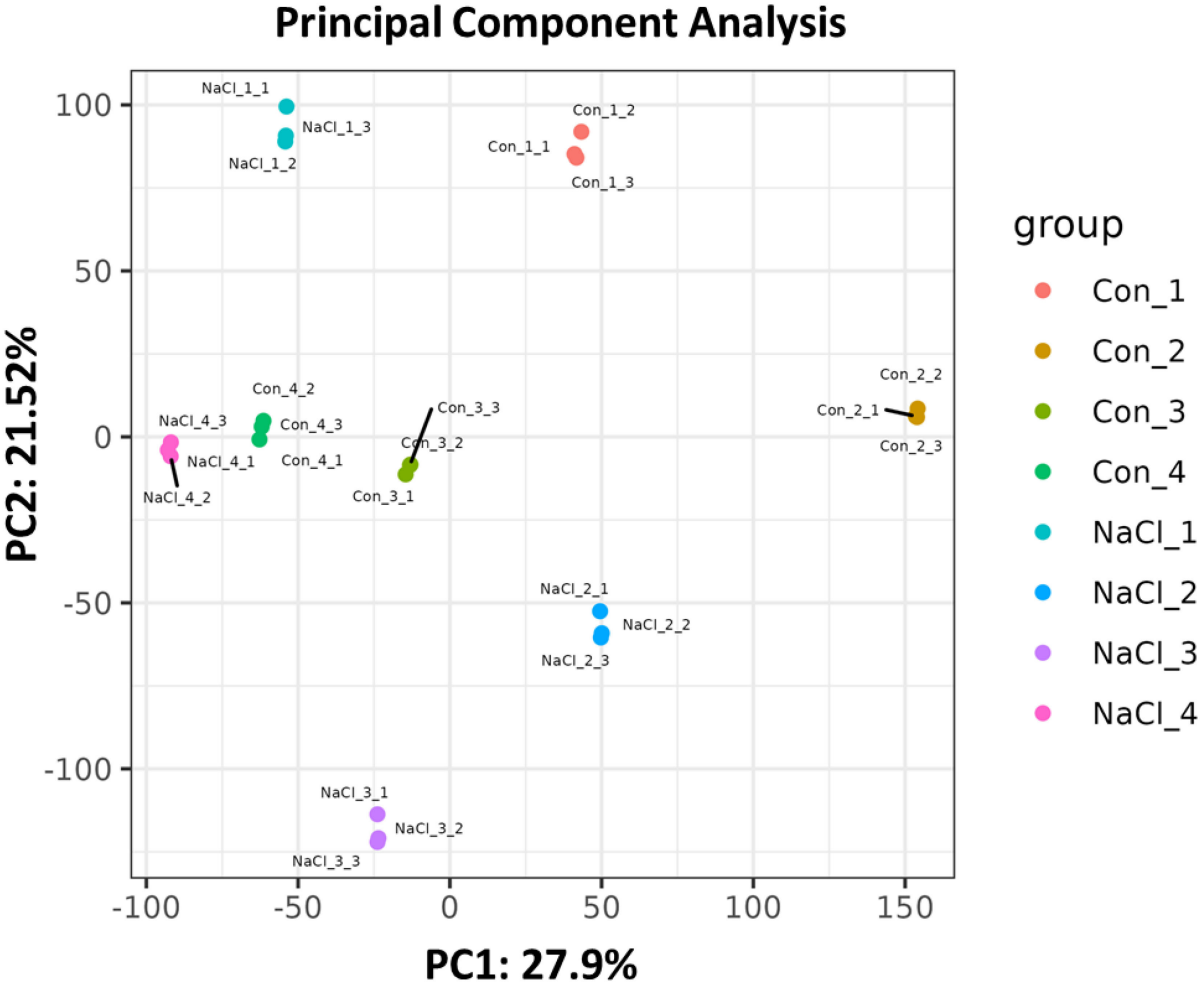
The principal component analysis according to the transcriptome samples. NaCl_1, NaCl_2, NaCl_3 and NaCl_4 represented the samples treated with 200 mM NaCl for 0, 2, 4, and 6 days. Con_1, Con_2, Con_3, and Con_4 represented the untreated samples for 0, 2, 4, and 6 days. Screening of differentially expressed genes (DEGs) among samples.

To investigate the relationship and correlations between each sample, principal component analysis (PCA) and correlation were performed, respectively. As shown in **Supplementary Data Sheet 2**, the Pearson correlation coefficient analysis indicated that the biological repeat R values for all groups exceeded 0.84, approaching 1. As shown in **Figure 2**, there were significant differences in the principal components across all groups. On the other hand, samples from other treatments had increased dispersion, which may indicate that there were more differentially expressed genes (DEGs) in each treatment.

**Figure 3:**
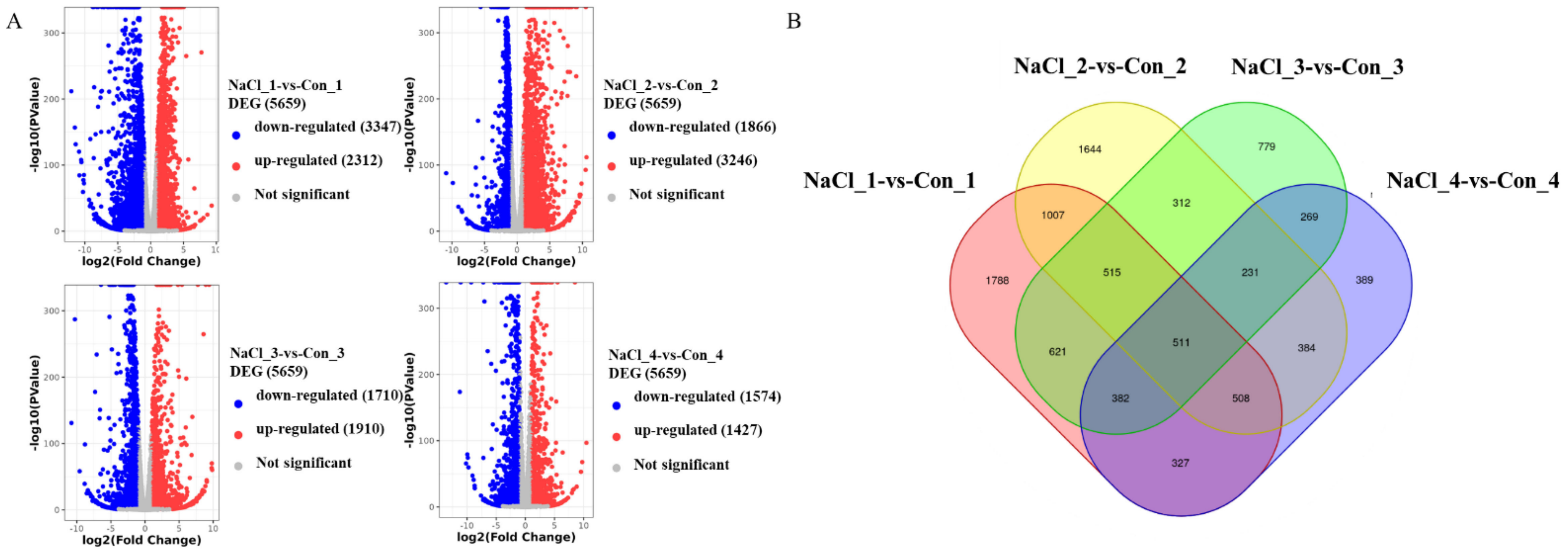
The volcano map analysis of differentially expressed genes (DEGs) **(A)** and the Venn map **(B)** analysis between the treatment group and the control group.

To identify differentially expressed genes (DEGs) among the samples, analyses were conducted for four paired groups: NaCl_1 vs. Con_1 (5659 DEGs), NaCl_2 vs. Con_2 (5112 DEGs), NaCl_3 vs. Con_3 (3620 DEGs), and NaCl_4 vs. Con_4 (3001 DEGs) (**Figure 3A**). Moreover, the NaCl_1 vs. Con_1 comparison revealed 2084 DEGs, with 2312 genes upregulated and 3347 downregulated, while the NaCl_2 vs. Con_2 comparison identified 5112 DEGs, comprising 3246 upregulated and 1866 downregulated genes. 3620 DEGs were found in the NaCl_3 vs. Con_3 comparison (1910 upregulated and 1710 downregulated), and 3001 DEGs were observed in the NaCl_4 vs. Con_4 comparison (1427 upregulated and 1574 downregulated), respectively. Additionally, the Venn diagram analysis (**Figure 3B**) illustrated that there were 511 co-expressed DEGs across the four paired groups, which might indicate these DEGs functioned more importantly for *A. camelorum* in response to salt stress.

Furthermore, the identification of DEGs across the four paired comparison groups provides valuable insights into the molecular response of *A. camelorum* to salt stress. The substantial number of DEGs, ranging from 3,001 to 5,659 in each comparison, indicated widespread transcriptional reprogramming in response to saline conditions. Notably, the NaCl_2 vs. Con_2 comparison exhibited the highest number of DEGs (5,112), suggesting this stage may represent a critical point of stress response or adaptation. The upregulation and downregulation patterns observed reflect dynamic adjustments in gene expression, potentially involving pathways related to osmoprotection, ion transport, antioxidant activity, and stress signaling. For example, the 511 co-expressed DEGs identified across all groups likely represent core components of the salt stress response, underpinning essential mechanisms that enable *A. camelorum* to tolerate high salinity. These conserved DEGs may include genes involved in maintaining cellular homeostasis, detoxifying reactive oxygen species, or modulating gene regulatory networks.

Biologically, these gene expression changes highlight the plant’s multifaceted strategy to perceive and adapt to salt stress, which can inform targeted approaches in breeding or conservation programs. By focusing on these key DEGs, especially the conserved ones, researchers can identify potential biomarkers or genetic targets to enhance salt tolerance. Overall, understanding these transcriptional adjustments advances our knowledge of *A. camelorum*’s resilience mechanisms and provides a foundation for future efforts to improve its survival in saline environments.

### 3.4 The GO annotation of DEGs among the samples

**Figure 4:**
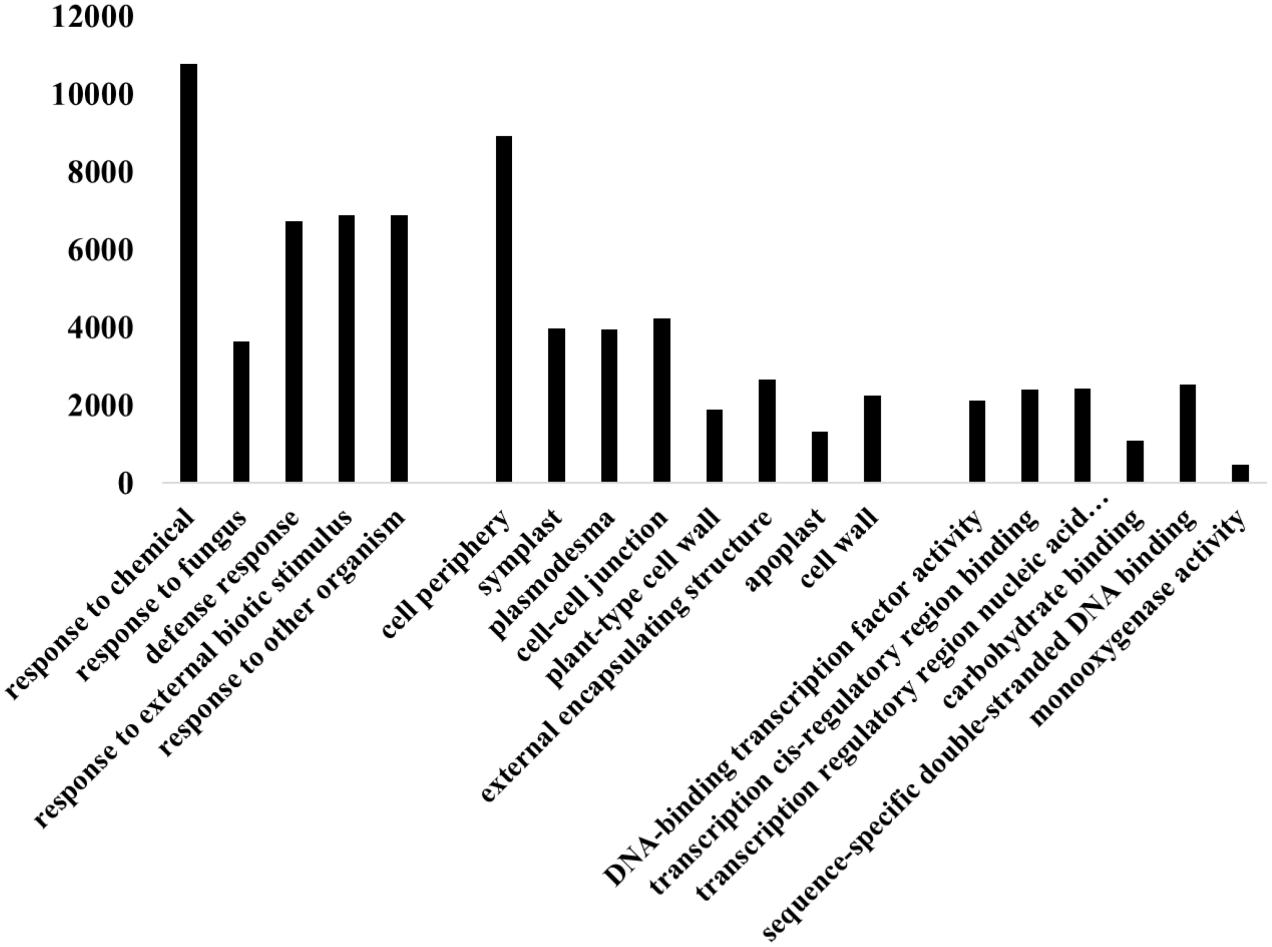
The GO annotation of DEGs in the comparable group NaCl_2 vs. Con_2.

To explore the biological functions of the identified differentially expressed genes (DEGs) in the four pair groups, GO annotation analysis was conducted. Here, we took the comparable group NaCl_2 vs. Con_2 as an example. As illustrated in **Figure 4**, we identified DEGs associated with the top 14 metabolic pathways. In terms of biological processes, the focus was primarily on pathways related to the response to chemicals (GO:0042221), among others. For cellular components, the emphasis was on pathways linked to the cell periphery (GO:0071944) and similar areas. Regarding molecular function, the main concentration was on pathways involving monooxygenase activity (GO:0004497).

### 3.5 KEGG enrichment analysis of DEGs among samples

**Figure 5:**
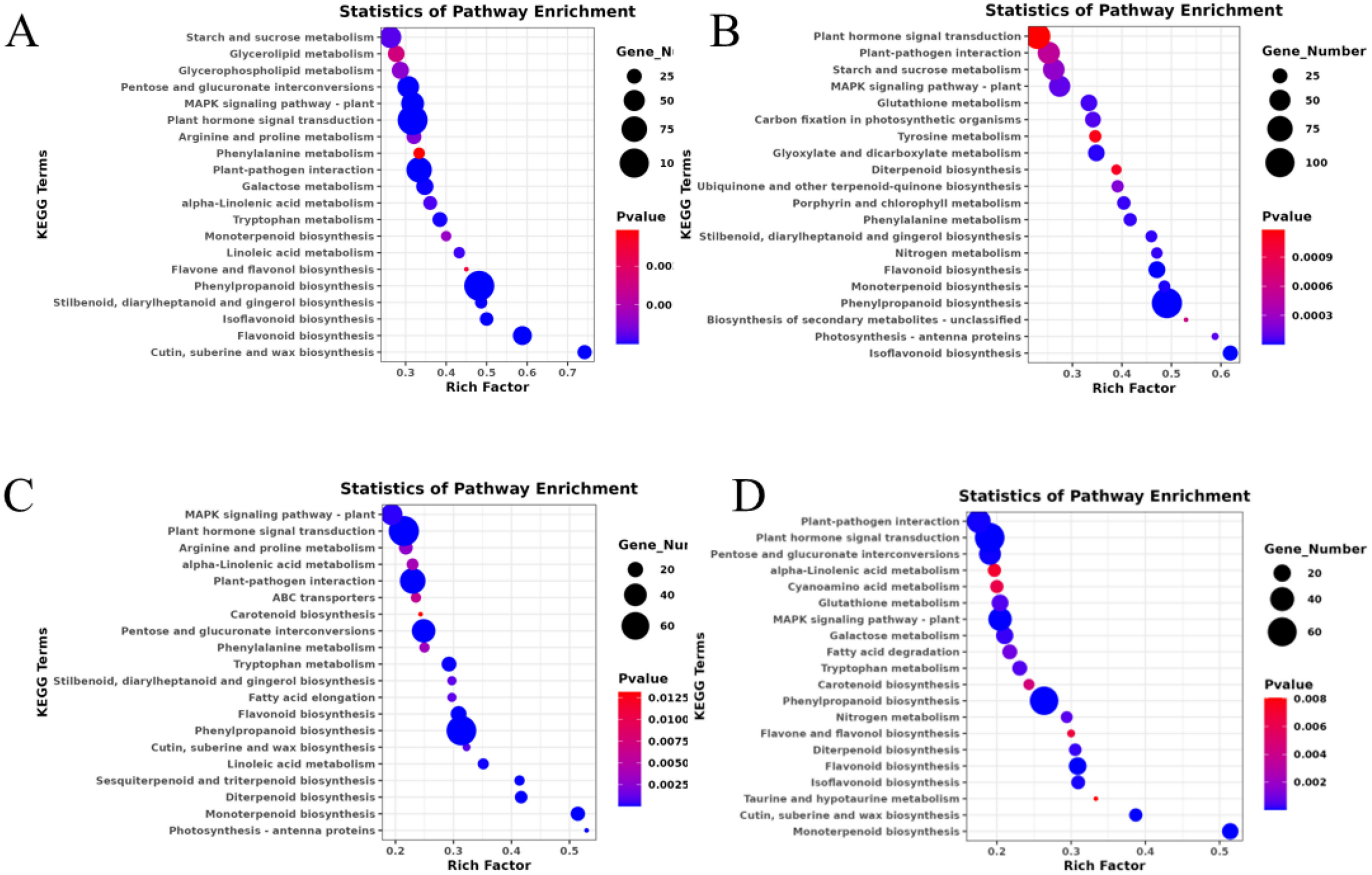
KEGG enrichment analysis of DEGs. The top 20 KEGG enrichment pathways were analyzed in four groups, including NaCl_1 vs. Con_1 **(A)**, NaCl_2 vs. Con_2 **(B)**, NaCl_3 vs. Con_3 **(C)**, and NaCl_4 vs. Con_4 **(D)**. The vertical axis represents the pathway name, the horizontal axis represents the rich factor, the size of dots in the pathway represents the number of DEGs, and the p-adjust value is reflected by the color of the dots.

KEGG enrichment analyses were performed to identify the important pathways associated with salt stress and to improve our comprehension of the biological processes involved in the various treatments. Four groups of the top 20 KEGG enrichment pathways were chosen for the study, shown in **Figure 5**. Among them, plant-pathogen interaction, plant hormone signal transduction, MAPK signaling pathway, phenylpropanoid biosynthesis, flavonoid biosynthesis, and monoterpenoid biosynthesis are the common main pathways among the four comparative groups, which indicate these DEGs or metabolites played an important role in response to salt stress of *A. camelorum*.

### 3.6 Metabolome analyses of samples

**Figure 6:**
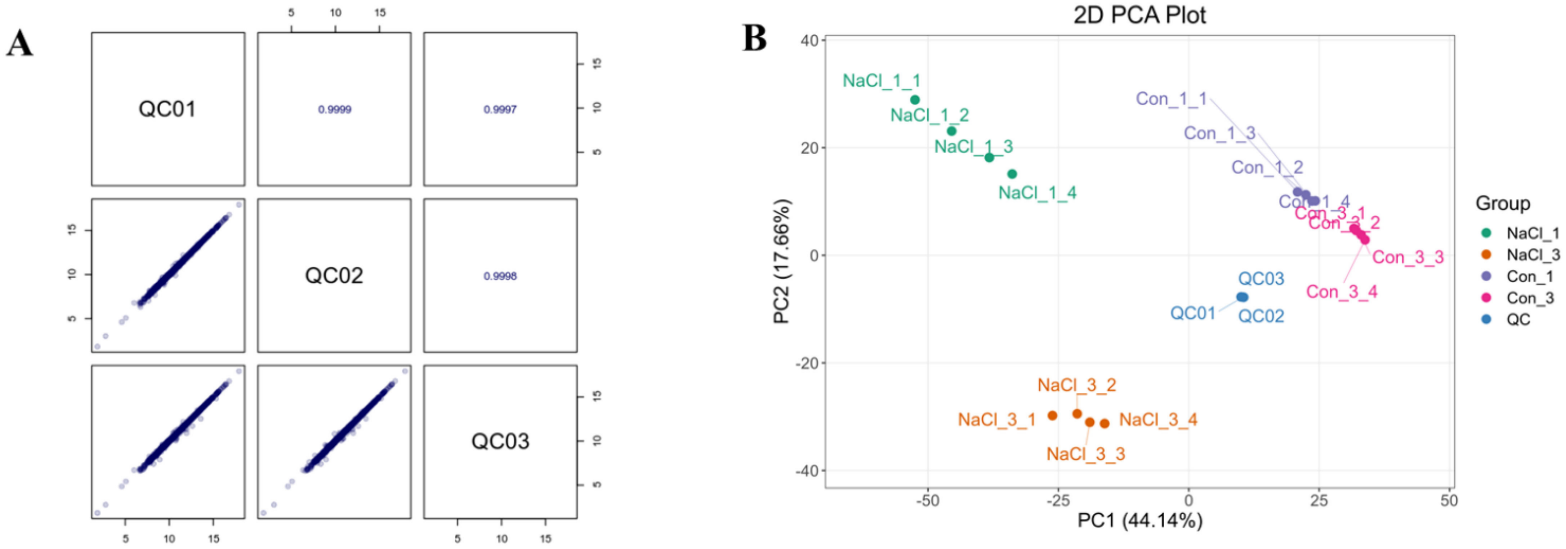
The Pearson correlation **(A)** and principal component analysis **(B)** plot according to the metabolome samples. NaCl_1 and NaCl_3 represented the samples treated with 200 mM NaCl for 0 and 4 days. Con_1 and Con_3 represented the untreated samples for 0 and 4 days.

To further elucidate which metabolites were affected under 200 mM NaCl treatment on *A. camelorum*, the metabolome analysis was conducted at two time points (NaCl and NaCl_3). Firstly, the correlation analysis and PCA analysis were used to assess the connections among the treated samples. A high degree of consistency was shown by the close alignment of the correlation plot for each sample’s biological replicates, as shown in **Figure 6A**. The Pearson correlation coefficient r approached 1, indicating that the experimental data were reliable. Furthermore, the PCA findings showed that the samples were dispersed and unique throughout the plot, indicating that these treatment groups probably had higher levels of differentially accumulated metabolites (DAMs) (**Figure 6B**). Thus, the separation trend of principal components among the four groups was pronounced, indicating a significant difference.

### 3.7 The classification and heatmap analysis of the metabolites

**Figure 7:**
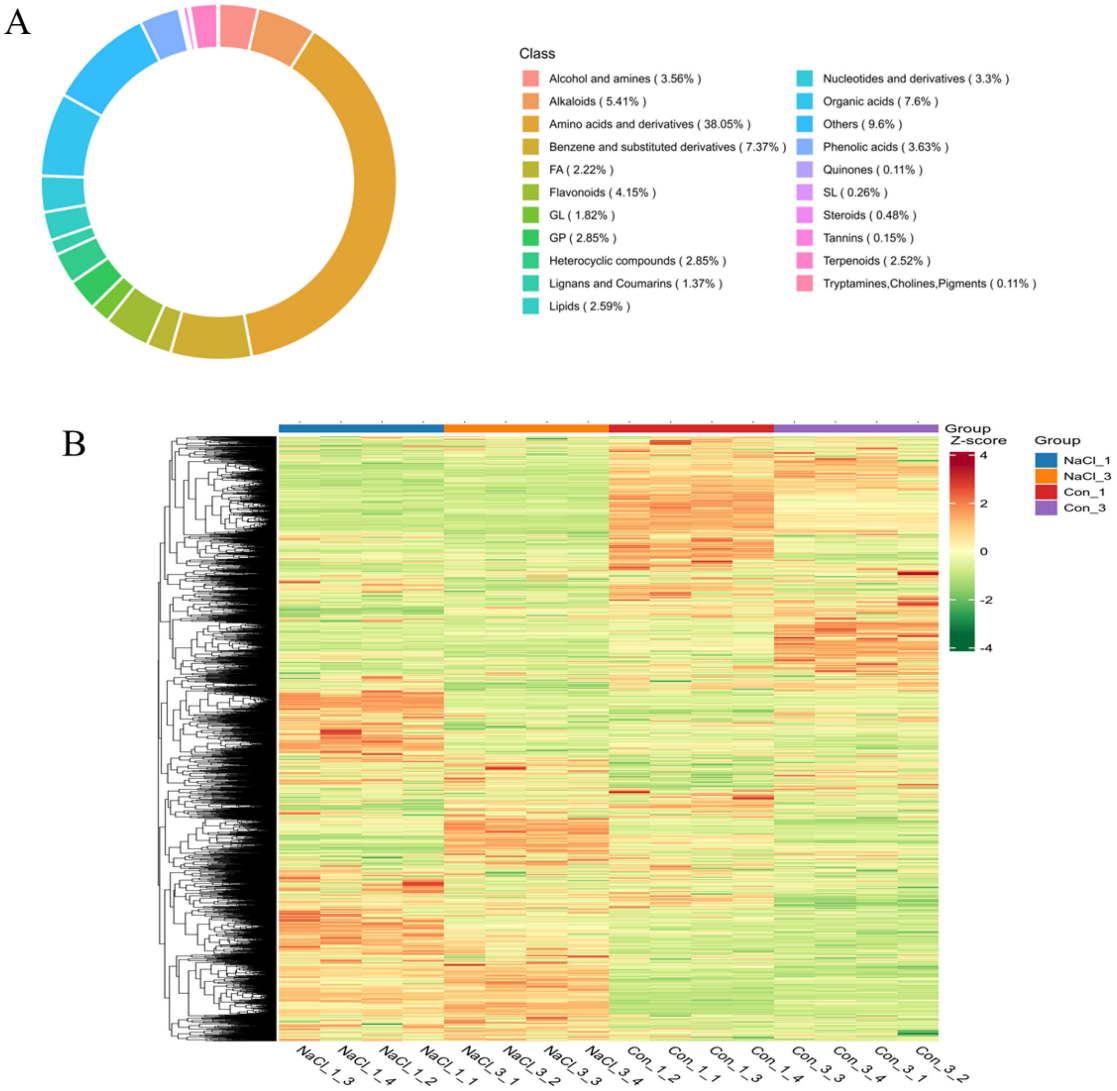
The circular diagram of metabolite categories **(A)** and heatmap analysis of metabolites **(B)** among NaCl-treated and untreated samples.

The analysis of metabolite composition reveals the overall landscape of biochemical changes in *A. camelorum* under salt stress. By analyzing the proportion of metabolite composition, the distribution of major metabolites in the sample can be examined as a whole. **Figure 7A** shows a circular diagram of metabolite categories on different platforms. Among them, 21 clusters were formed, and the main metabolites clustered into amino acids and derivatives (38.05%), organic acids (7.6%), benzene and substituted derivatives (7.37%), etc. groups. This distribution indicates that amino acids and their derivatives were predominant components of *A. camelorum*’s metabolome and likely play key roles in the stress response. Furthermore, the accumulation of 2700 metabolites was analyzed, and they behaved differently among NaCl-treated and untreated samples (**Figure 7B**), indicating that the main differentially accumulated metabolites existed among them, which were affected by salt stress. Further, these showed distinct behaviors between NaCl-treated and untreated samples, underscoring that salt stress triggered significant shifts in metabolic profiles. In relation to tolerance, the prominent accumulation of amino acids and derivatives suggested that *A. camelorum* synthesizes osmolytes like proline, glycine betaine, and other compatible solutes to maintain cellular osmotic balance and protect cellular components. The changes in organic acids and benzene derivatives may reflect adjustments in energy metabolism and secondary metabolite pathways involved in stress mitigation and defense. In short, these metabolite alterations were integral to the plant’s adaptive strategies, helping to stabilize proteins, membranes, and enzymes under salt stress, thereby enhancing survival and resilience. Understanding these metabolite profiles can aid in identifying key biochemical pathways that underpin salt tolerance, informing future efforts to improve stress resilience in *A. camelorum* plants.

### 3.8 The analysis of differentially accumulated metabolites (DAMs) among samples

**Figure 8:**
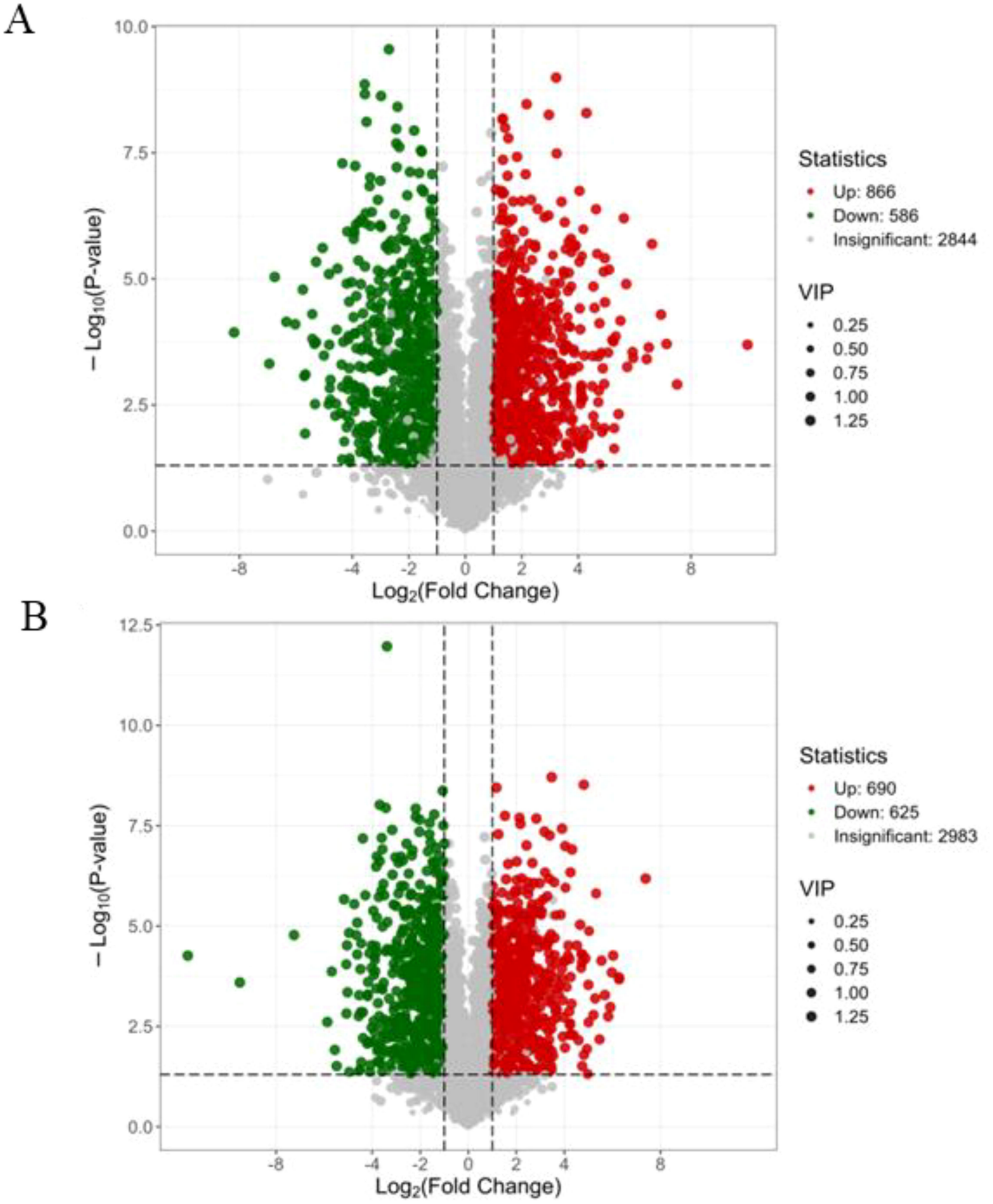
The volcano map analysis of differentially accumulated metabolites (DAMs) in NaCl_1 vs. Con_1 **(A)** and NaCl_3 vs. Con_3 **(B)** groups.

To identify differentially accumulated metabolites (DAMs) among the samples, the volcano map analysis was conducted for the samples. The 866 and 586 were up and down accumulated in metabolites of the NaCl_1 vs. Con_1 group, respectively (**Figure 8A**). While 690 and 625 were up and down accumulated in metabolites of NaCl_3 vs. Con_3, respectively (**Figure 8B**), reflecting ongoing metabolic adjustments as the plant adapts to sustained salinity conditions. Similarly, 2844 and 2983 DAMs were insignificant among the treated samples, respectively. Furthermore, the first twenty up or down DAMs in NaCl_1 vs. Con_1 and NaCl_3 vs. Con_3 groups were shown in **Supplementary Data Sheets 3**; **4**; they were almost all amino acids, carbohydrates, flavonoids, etc.

Among the DAMs, in the group of NaCl_1 vs. Con_1, the amino acids and derivatives, FA, heterocyclic compounds, and flavonoids were significantly upregulated (**Table 2**). In the group of NaCl_3 vs. Con_3, the amino acids and derivatives, heterocyclic compounds, and flavonoids were significantly accumulated, while the accumulation of flavonoids was the highest (**Table 3**). Thus, the flavonoids could be the key DAMs that were affected by salt stress in *A. camelorum*.

**Table 2:** The main DAMs in the group of NaCl_1 vs. Con_1.

Index	Class	VIP	FDR	Fold_Change	Log2FC	Type
MW0106978	Amino acids and derivatives	1.31008003	0.000441254	3.222130614	1.68801498	up
MW0141380	FA	1.122736784	0.09637847	3.651942353	1.86866399	up
MW0054028	Others	1.222915618	0.002776264	0.170928382	-2.5485361	down
MW0048964	Others	1.313930097	0.000326955	0.100303879	-3.3175507	down
MW0109185	Amino acids and derivatives	1.309281803	0.000261417	2.59303618	1.37464234	up
MW0058015	GP	1.285182067	0.000432466	3.294714658	1.72015353	up
MW0128381	Heterocyclic compounds	1.308155063	0.00011311	30.69795537	4.94007066	up
MW0132722	Flavonoids	1.127039468	0.036961425	5.411089718	2.43591916	up
MW0106536	Amino acids and derivatives	1.258810264	0.016432842	0.401342866	-1.3170928	down
MW0139812	Heterocyclic compounds	1.247502754	0.009744891	0.024971683	-5.3235632	down

**Table 3:** The main DAMs in the group of NaCl_3 vs. Con_3.

Index	Class 1	VIP	FDR	Fold_Change	Log2FC	Type
MW0106978	Amino acids and derivatives	1.32020316	0.000110918	3.264024236	1.70665177	up
MW0111217	Alcohol and amines	1.32361349	0.000234766	0.258174955	-1.953579038	down
MW0106789	Amino acids and derivatives	1.28405677	0.006890334	12.49678371	3.643484931	up
MW0110760	Alcohol and amines	1.274033579	0.000709378	0.317579953	-1.65480825	down
MW0054028	Others	1.263186302	0.000478067	0.168237549	-2.571428361	down
MW0048964	Others	1.326683759	3.59E-05	0.112478955	-3.152273002	down
MW0014589	Lipids	1.132175277	0.010203599	0.497753936	-1.006495372	down
MW0144984	Others	1.108290968	0.145979728	0.420366645	-1.250279896	down
MW0128381	Heterocyclic compounds	1.323899495	0.000110918	6.091323842	2.606755807	up
MW0132722	Flavonoids	1.183203454	0.000125594	8.196573728	3.035020971	up

### 3.9 KEGG enrichment analysis of DAMs

**Figure 9:**
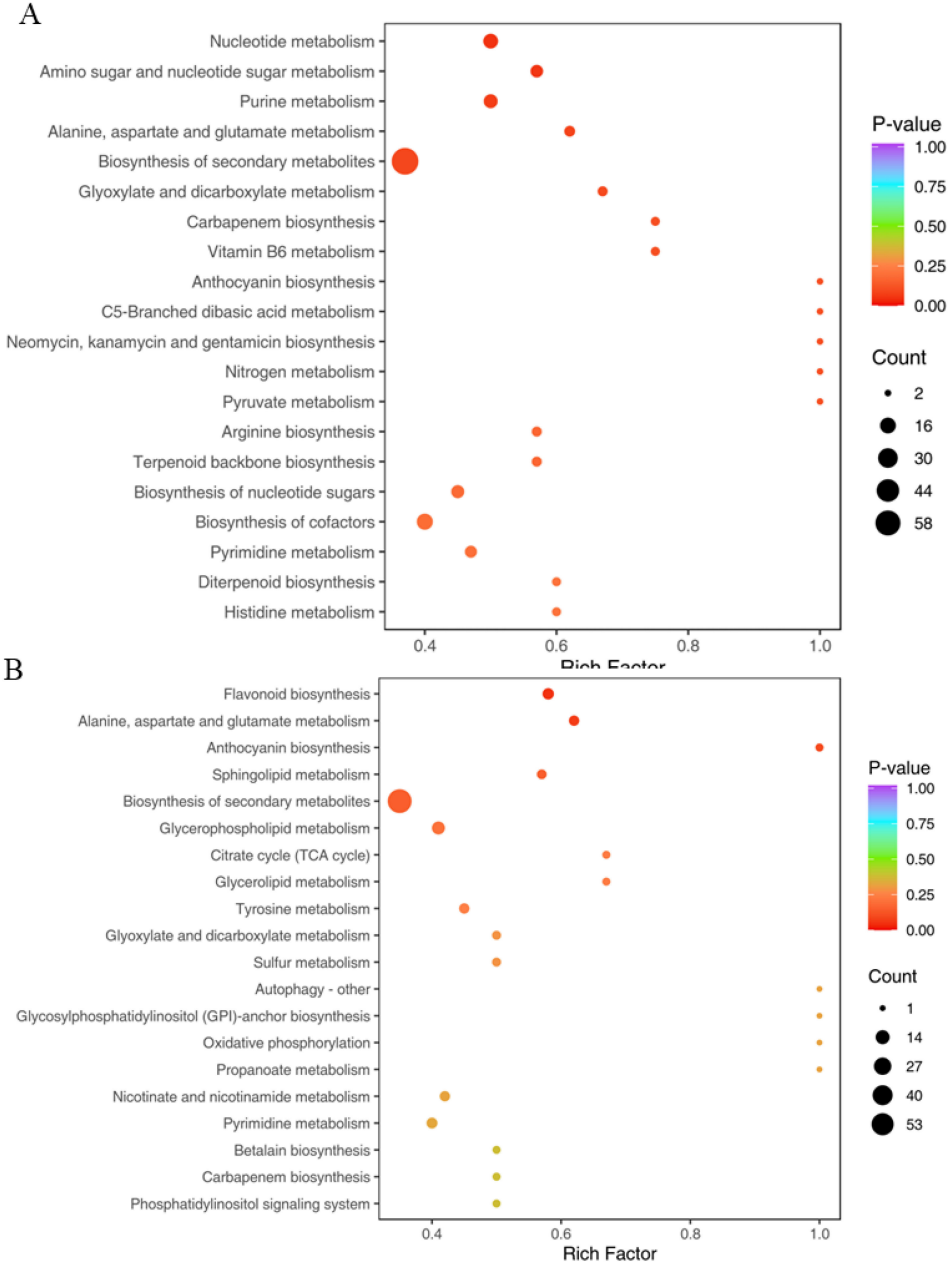
KEGG enrichment analysis of DAMs. The top 20 KEGG enrichment pathways were analyzed in two groups, including NaCl_1 vs. Con_1 **(A)** and NaCl_3 vs. Con_3 **(B)**. The vertical axis represents the pathway name, the horizontal axis represents the rich factor, the size of dots in the pathway represents the number of DAMs, and the p-adjust value is reflected by the color of the dots.

KEGG enrichment analyses of DAMs were performed to identify the important pathways associated with salt stress and to improve our comprehension of the biological processes involved in the various treatments (**Figure 9**). It was found that the same pathways enriched with metabolites in both groups include alanine, aspartate, and glutamate metabolism, biosynthesis of secondary metabolites, glyoxylate and dicarboxylate metabolism, carbapenem biosynthesis, and anthocyanin biosynthesis, as shown in **Figures 9A, B**, which might indicate these pathway metabolites were significantly affected by salt stress.

### 3.10 Assessment of the comparative relationship between transcriptome and metabolome

**Figure 10:**
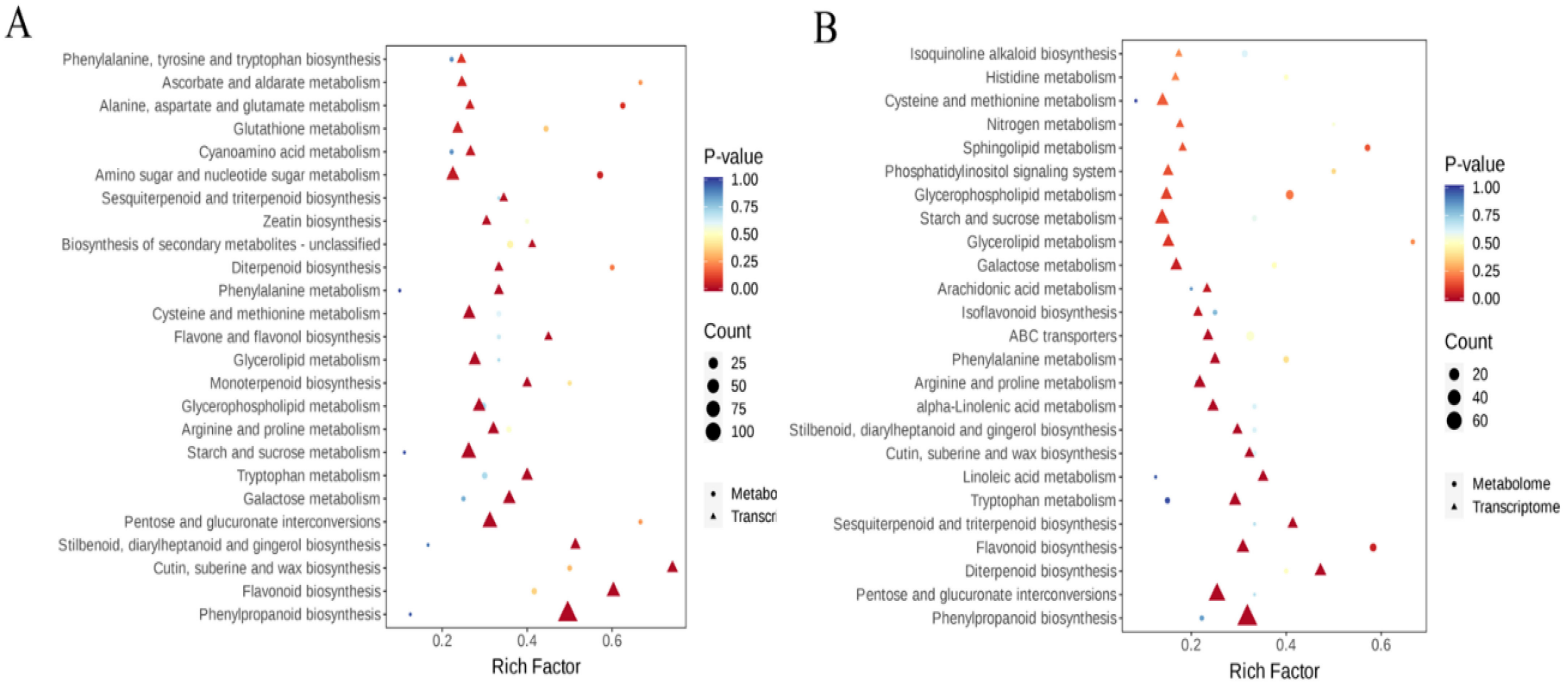
Transcriptome and metabolome combined KEGG enrichment analysis. The top 25 KEGG enrichment pathways were analyzed in two groups of NaCl_1 vs. Con_1 **(A)** and NaCl_3 vs. Con_3 **(B)**. The vertical axis represents the pathway name, the horizontal axis represents the rich factor, the size of dots and triangles in the pathway represents the number of DAMs and DEGs, and the p-adjust value is reflected by the color of the dots and triangles.

Integrated transcriptome and metabolome analyses were conducted to identify the key pathways associated with differentially expressed genes (DEGs) and differentially accumulated metabolites (DAMs). To explore the connections between genes and metabolites associated with salt stress tolerance, differentially regulated genes and differentially abundant metabolites from the two comparison groups (salt treatment vs. control treatment) were concurrently mapped to KEGG pathways. By using the pathways co-enriched by the two omics, we select the top 25 pathways with a *P*-value identified for salt treatments. The common enrichment pathways of the two groups were sesquiterpenoid and triterpenoid biosynthesis, diterpenoid biosynthesis, phenylalanine metabolism, cysteine and methionine metabolism, glycerophospholipid metabolism, arginine and proline metabolism, and tryptophan metabolism (**Figures 10A, B**). Among them, the pathways of flavonoid biosynthesis were the most significant.

### 3.11 Identification of DEGs associated with the flavonoid biosynthesis metabolites

**Figure 11:**
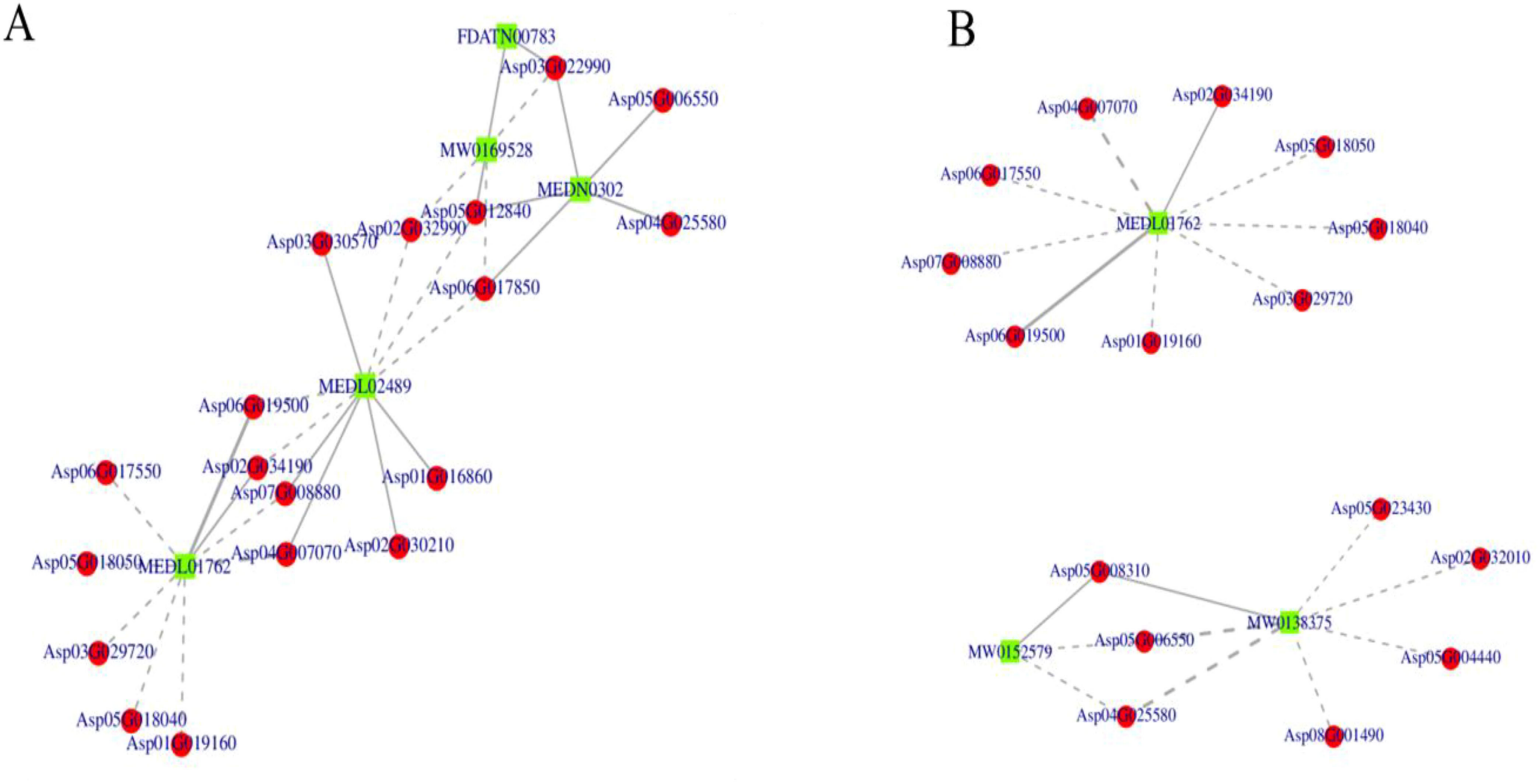
Identification of DEGs associated with the flavonoid biosynthesis metabolites in two groups of NaCl_1 vs. Con_1 **(A)** and NaCl_3 vs. Con_3 **(B)**. The DEGs (*Asp04G007070, Asp04G008880, Asp06G017850, Asp02G032990, Asp05G012840, Asp03G022990, Asp05G008310, Asp05G006550*) were regulated, which were closely related to the accumulation of flavonoid biosynthesis metabolites, such as MEDL1762, MEDL02489, and MEDN0302, etc., in both groups.

Flavonoids function as plant antitoxins or antioxidants by effectively eliminating ROS and shielding plants from various biotic and abiotic stresses, including ultraviolet radiation, cold stress, and drought (Muhammad et al., 2025; Wang X. et al., 2020; Zhu et al., 2024). This suggests that flavonoids may be essential mediators in the plant’s adaptive strategies to cope with environmental challenges. Flavonoid biosynthesis primarily occurs via the phenylpropanoid metabolic pathway, a complex and tightly regulated process (Muhammad et al., 2022, 2023a). External environmental factors directly influence the expression of genes related to enzymes responsible for flavonoid production, as well as the transcription factors that regulate this process (Muhammad et al., 2024, 2023b). The correlation network was analyzed to further identify the key differentially expressed genes associated with flavonoid biosynthesis metabolites under salt stress As shown in **Figure 11** and **Supplementary Data Sheet 4**, the DEGs (*Asp04G007070, Asp04G008880, Asp06G017850, Asp02G032990, Asp05G012840, Asp03G022990, Asp05G008310, Asp05G006550*) were regulated which were closely related with the accumulation of flavonoid biosynthesis metabolites, such as MEDL1762, MEDL02489, and MEDN0302 etc. in both groups, which indicated the salt stress on *A. camelorum* could mainly affect the flavonoid biosynthesis metabolites by these DEGs.

### 3.12 The expression analysis of flavonoid biosynthesis-related genes

**Figure 12:**
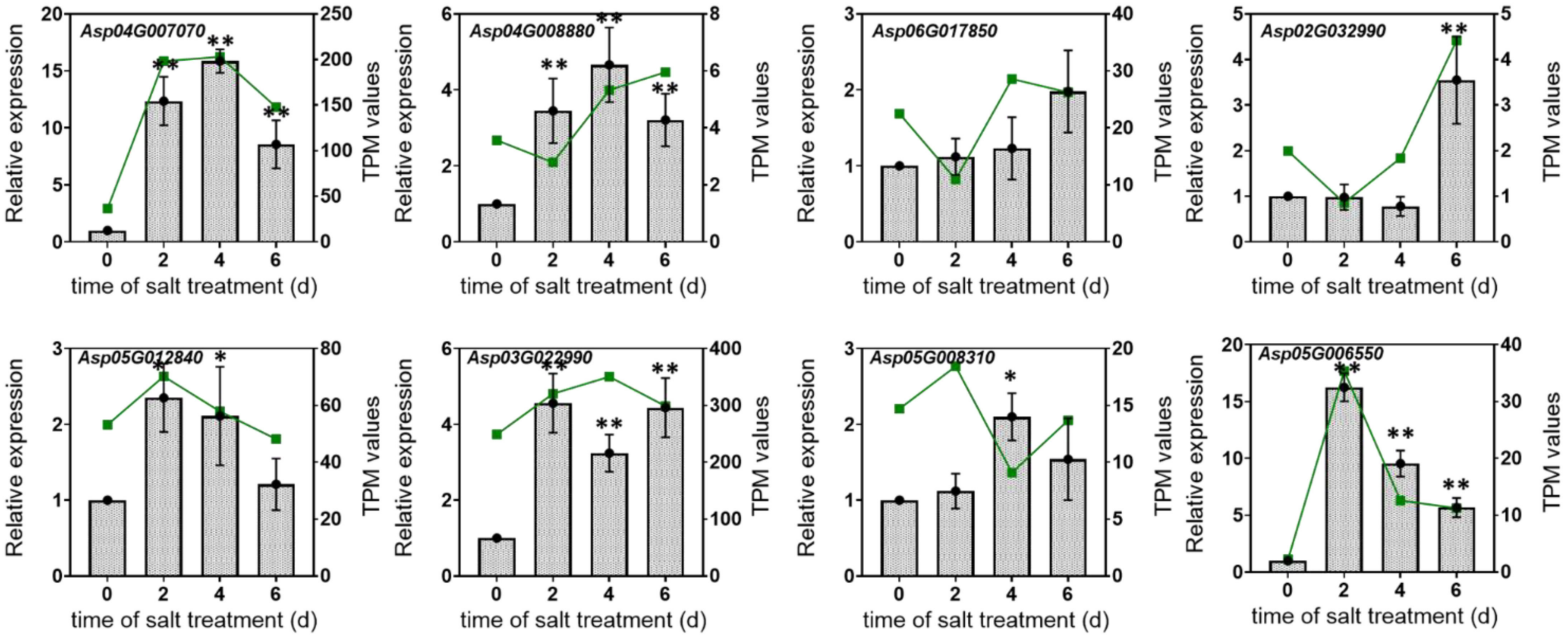
Verification of the expression of flavonoid biosynthesis-related genes in response to salt stress by qPCR analysis. The column chart represented the expression level by qPCR analysis, while the green line indicated the changes in TPM values. * and ** represent the different significance at *p* < 0.05 and *p* < 0.01 level, respectively. The calculation of different significance was the salt-treated time (2, 4, 6 h) compared to the 0 h time point.

To further verify the expression level of the above flavonoid biosynthesis-related genes in response to salt stress, the qPCR analysis was performed. As shown in **Figure 12**, the expression level of *Asp04G007070, Asp04G008880, Asp06G017850, Asp02G032990, Asp05G012840, Asp03G022990, Asp05G008310*, and *Asp05G006550* was significantly induced after salt stress treatment, and the changing trend was almost consistence with their TPM values. The results further demonstrated that the accumulation of flavonoids contributed to the tolerance of salt stress in *A. camelorum*.

## 4 Discussion

Soil salinity stress has emerged as a significant abiotic challenge to plant species. Salt stress, being a major environmental factor, affects the global distribution of plants, constraining their growth and productivity while posing a threat to the plant biodiversity of the area. To enhance plant resistance to salt stress, it is essential to identify key mechanisms of plant response and tolerance to salinity (Y. Zhang et al., 2019). Thus, we investigated the transcriptomic and metabolic changes in *A. camelorum* under salinity stress over a period of six days. This allowed us to identify key genes and metabolites that are involved in the underlying metabolic pathways of salt tolerance in *A. camelorum*.

Moreover, the detrimental impact of salinity (NaCl) on plant growth has been observed in a variety of species, including wheat, maize, rice, and various other shrubs and trees (Das and Roychoudhury, 2014; Huang et al., 2021; Sun et al., 2022; D. Zhang et al., 2023). Consistent with these reports, our current study found significant decreases in the number of leaves, shoot length, main root length, and lateral root number of *A. camelorum*, indicating that salt stress has caused a more extensive effect on *A. camelorum* growth. It has also been found that high salt concentrations have an osmotic effect on plant cells (Munns and Tester, 2008; Roy et al., 2014). When subjected to salt stress, plants quickly face osmotic pressure that constricts cell expansion in root tips and young leaves, leading to a significant reduction in stomatal conductance (Liu et al., 2019; Munns and Tester, 2008). Numerous plants have the ability to accumulate compatible solutes to sustain stable osmotic pressure, helping to safeguard membranes and proteins from degradation through osmotic regulation (Bohnert et al., 1995; Türkan and Demiral, 2009). In agreement with prior studies, our investigation showed that the majority of amino acids and their derivatives, organic acids, and benzene and substituted derivatives, among other groups, exhibited significant accumulation, highlighting the main differentially accumulated metabolites that were influenced by salt (NaCl) stress. The number of DEGs with up- and downregulation may also have a pivotal role in response to salt stress. Previous studies have investigated the expression of DEGs in salt-treated plant species, with varying results: up- and downregulation observed across different plant species (Shu et al., 2022; Xie et al., 2021; Zhang et al., 2023). Whereas in our analysis, a total of 3620 genes in the two comparing groups (NaCl_3 vs Con_3) showed significant differences in expression, with 1910 genes upregulated and 1710 downregulated. On the other hand, the comparison between NaCl_4 vs Con_4 revealed a total of 3001 differentially expressed genes, of which 1427 were upregulated and 1574 downregulated, emphasizing the significant role of these DEGs in response to salt stress of *A. camelorum*. Similarly, the metabolites produced by plants function as free radical scavengers, osmoprotectants, and signaling molecules (Shiade et al., 2024). Up and down accumulation of metabolites has an important role in plant stress response and tolerances (Kumar et al., 2021; Niu et al., 2024). In this study, the 866 and 586 were up and down accumulated metabolites of NaCl_1 vs. Con_1 group, respectively. While 690 and 625 were up and down accumulated in metabolites of NaCl_3 vs. Con_3, respectively, highlighting their important potential role in mitigating the NaCl stress.

Previously, it has also been identified that the accumulation of metabolites such as proline and inositol in barley roots plays a crucial role in mitigating salt stress (Shen et al., 2018). These metabolites enhance antioxidant capacity, helping to neutralize reactive oxygen species (ROS) and reducing energy expenditure during salt stress (Shen et al., 2018; Wu et al., 2013). This study reveals that under NaCl stress, various metabolic pathways are significantly enriched, including those related to sesquiterpenoid and triterpenoid biosynthesis, diterpenoid biosynthesis, phenylalanine metabolism, cysteine and methionine metabolism, glycerophospholipid metabolism, arginine and proline metabolism, and tryptophan metabolism. These findings underscore the importance of these pathways in helping *A. camelorum* cope with salt stress. The KEGG database facilitates pathway-based analysis, enabling researchers to systematically explore the intricate biological processes and networks associated with stress responses (Jin et al., 2021). Herein are the top 20 KEGG enrichment pathway analyses across the four groups. Within these pathways, four families represent significant enrichment, which is related to stress response, including plant-pathogen interaction, plant hormone signal transduction, MAPK signaling pathway, phenylpropanoid biosynthesis, monoterpenoid biosynthesis, and flavonoid biosynthesis, highlighting the *A. camelorum* defense mechanisms against stress, particularly involving potential interactions with NaCl stress.

Flavonoids are secondary metabolites known for their antioxidant properties, which enable plants to effectively scavenge reactive oxygen species (ROS) and inhibit their formation (Di Ferdinando et al., 2012). Furthermore, flavonoids have beneficial effects on environmental stress and serve various functions (Häusler et al., 2014). In addition to their antioxidant roles, various mechanisms and sites of action have been suggested for flavonoids in enhancing plant stress tolerance (Shomali et al., 2022). These compounds are generally specific to certain species (Harborne and Williams, 2000; Khalid et al., 2019), and their biosynthesis is influenced by the plant species, developmental stage, and the type of stress experienced (Samanta et al., 2011; Taulavuori et al., 2016). In this study, we observed that the expression levels of key genes associated with the flavonoid biosynthesis pathway influenced seed germination under salt stress. It has also been observed that the expression of genes involved in flavonoid biosynthesis, as well as the transcription factors that regulate this process, is directly affected by external environmental factors or stresses (N. Muhammad et al., 2025; Y. Wang et al., 2024b). Valifard et al. showed that a concentration of 100 mM NaCl markedly increased the expression of the key gene in the flavonoid pathway encoding phenylalanine ammonia-lyase- PAL in the leaves of two medicinal *Salvia* species, elevating it by 12 to 18 times and consequently boosting PAL activity (Valifard et al., 2015). In the present study, the expression levels of key genes (*Asp04G007070, Asp04G008880, Asp06G017850, Asp02G032990, Asp05G012840, Asp03G022990, Asp05G008310, Asp05G006550*) involved in the flavonoid pathway were significantly higher in the 100 mM and 200 mM treatment groups compared to the control. This suggests that these genes were highly expressed in response to 100 mM and 200 mM, likely due to the effects of salt stress. Thus in the current study, the regulated DEGs were found to be closely associated with the accumulation of metabolites involved in flavonoid biosynthesis, including MEDL1762, MEDL02489, and MEDN0302. This suggests that salt stress in *A. camelorum* primarily influences flavonoid biosynthesis through the regulation of these specific DEGs. Similarly, the pathways involved in flavonoid biosynthesis stand out as the most significant.

## 5 Conclusions

We explored changes in gene expression and metabolites under salinity conditions in *A. camelorum* seedlings using integrated physiological, metabolomic, and transcriptomic approaches. Our results revealed that the treatments with 100 mM and 200 mM NaCl resulted in a decrease in the number of leaves, shoot lengths, main root length, and the number of lateral roots after six days. The study uncovered molecular mechanisms regulating *A. camelorum* seedling response to salt stress. Specifically, the expression levels of several genes (*Asp04G007070, Asp04G008880, Asp06G017850, Asp02G032990, Asp05G012840, Asp03G022990, Asp05G008310, Asp05G006550*) associated with metabolite production were found to be key players in this response. These findings suggest that metabolite-mediated gene regulation is a crucial component *of A. camelorum*’s salt stress response. This knowledge could lead to the identification of potential indicators for improving salt tolerance in conservation programs. Further research is needed to fully understand the complete salt stress response pathway in *A. camelorum*.

## Data Availability

The data presented in the study are deposited in the in the NGDC database (https://ngdc.cncb.ac.cn) repository, accession number PRJCA051278 for metabolome and PRJCA051276 for transcriptome.
