# Supplementary material for: Integrative analysis of morphological, transcriptomic, and metabolomic approaches to uncover the function of flavonoids in the salt stress response of Alhagi camelorum
Source: Front Plant Sci. 2026 Jan 5;16:1678456. doi: 10.3389/fpls.2025.1678456 (PMC12812734; doi:10.3389/fpls.2025.1678456)
Supplement: Supplementary file 1 [file DataSheet1.docx]

Supplementary data 1 The quality analysis of transcriptome sequencing data

| Sample Name | Raw Reads | Adapter trimmed | Clean Rate | Q30% | GC% | Unique Mapped (%) | Multi Mapped (%) | Total Mapped (%) |
| --- | --- | --- | --- | --- | --- | --- | --- | --- |
| NaCl_1_1 | 23,689,363 | 23,685,779 | 99.98 | 95.29 | 44.05 | 22,138,966 (93.47) | 448,158 (1.89) | 22,587,124 (95.36) |
| NaCl_1_2 | 19,494,572 | 19,489,243 | 99.97 | 95.74 | 43.60 | 18,366,688 (94.24) | 363,757 (1.87) | 18,730,445 (96.11) |
| NaCl_1_3 | 19,575,957 | 19,571,815 | 99.98 | 95.86 | 43.57 | 18,518,446 (94.62) | 361,177 (1.85) | 18,879,623 (96.46) |
| NaCl_2_1 | 33,648,646 | 33,643,241 | 99.98 | 95.27 | 43.77 | 31,632,331 (94.02) | 582,643 (1.73) | 32,214,974 (95.75) |
| NaCl_2_2 | 24,545,563 | 24,539,153 | 99.97 | 95.27 | 43.34 | 23,263,134 (94.80) | 422,580 (1.72) | 23,685,714 (96.52) |
| NaCl_2_3 | 19,728,970 | 19,724,918 | 99.98 | 95.64 | 43.37 | 18,669,476 (94.65) | 337,783 (1.71) | 19,007,259 (96.36) |
| NaCl_3_1 | 26,650,699 | 26,647,304 | 99.99 | 95.19 | 43.85 | 25,138,570 (94.34) | 538,435 (2.02) | 25,677,005 (96.36) |
| NaCl_3_2 | 19,626,449 | 19,622,130 | 99.98 | 95.82 | 43.45 | 18,544,747 (94.51) | 398,035 (2.03) | 18,942,782 (96.54) |
| NaCl_3_3 | 25,141,742 | 25,136,229 | 99.98 | 95.04 | 43.46 | 23,721,419 (94.37) | 511,105 (2.03) | 24,232,524 (96.40) |
| NaCl_4_1 | 23,170,027 | 23,166,760 | 99.99 | 95.26 | 44.07 | 21,732,182 (93.81) | 424,879 (1.83) | 22,157,061 (95.64) |
| NaCl_4_2 | 23,171,933 | 23,166,521 | 99.98 | 95.65 | 43.88 | 21,859,310 (94.36) | 421,440 (1.82) | 22,280,750 (96.18) |
| NaCl_4_3 | 25,155,024 | 25,148,365 | 99.97 | 95.48 | 43.98 | 23,640,456 (94.00) | 457,178 (1.82) | 24,097,634 (95.82) |
| Con_1_1 | 26,262,054 | 26,259,249 | 99.99 | 95.27 | 44.16 | 24,770,849 (94.33) | 464,325 (1.77) | 25,235,174 (96.10) |
| Con_1_2 | 20,805,668 | 20,800,877 | 99.98 | 95.64 | 44.01 | 19,609,858 (94.27) | 369,141 (1.77) | 19,978,999 (96.05) |
| Con_1_3 | 24,729,474 | 24,723,905 | 99.98 | 95.47 | 44.07 | 23,301,372 (94.25) | 443,440 (1.79) | 23,744,812 (96.04) |
| Con_2_1 | 25,185,675 | 25,181,743 | 99.98 | 95.14 | 44.10 | 23,762,687 (94.36) | 435,589 (1.73) | 24,198,276 (96.09) |
| Con_2_2 | 25,702,266 | 25,694,965 | 99.97 | 95.28 | 44.00 | 24,140,195 (93.95) | 456,112 (1.78) | 24,596,307 (95.72) |
| Con_2_3 | 25,363,566 | 25,357,684 | 99.98 | 95.24 | 44.02 | 23,876,927 (94.16) | 443,740 (1.75) | 24,320,667 (95.91) |
| Con_3_1 | 23,020,766 | 23,017,819 | 99.99 | 95.45 | 44.08 | 21,698,328 (94.27) | 437,183 (1.90) | 22,135,511 (96.17) |
| Con_3_2 | 26,607,757 | 26,599,516 | 99.97 | 95.19 | 44.08 | 24,963,743 (93.85) | 510,041 (1.92) | 25,473,784 (95.77) |
| Con_3_3 | 27,958,127 | 27,953,163 | 99.98 | 95.47 | 44.01 | 26,410,610 (94.48) | 526,493 (1.88) | 26,937,103 (96.37) |
| Con_4_1 | 23,539,431 | 23,536,512 | 99.99 | 94.93 | 43.99 | 22,236,476 (94.48) | 416,742 (1.77) | 22,653,218 (96.25) |
| Con_4_2 | 22,798,122 | 22,793,745 | 99.98 | 95.47 | 43.89 | 21,614,952 (94.83) | 401,606 (1.76) | 22,016,558 (96.59) |
| Con_4_3 | 23,649,235 | 23,645,150 | 99.98 | 96.11 | 43.82 | 22,410,900 (94.78) | 418,525 (1.77) | 22,829,425 (96.55) |

Note: NaCl_1， NaCl_2， NaCl_3 and NaCl_4 represented the samples treated with 200 mM NaCl for 0,2,4,6 days. Con_1, Con_2, Con_3 and Con_4 represented the untreated samples for 0,2,4,6 days.
