# Supplementary material for: Integrative analysis of morphological, transcriptomic, and metabolomic approaches to uncover the function of flavonoids in the salt stress response of Alhagi camelorum
Source: Front Plant Sci. 2026 Jan 5;16:1678456. doi: 10.3389/fpls.2025.1678456 (PMC12812734; doi:10.3389/fpls.2025.1678456)
Supplement: Supplementary file 2 [file DataSheet2.docx]

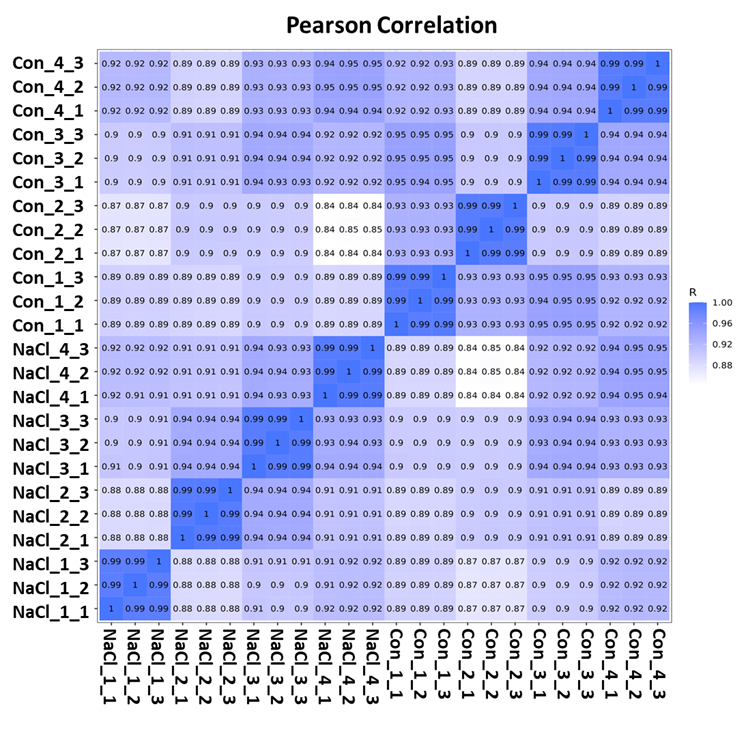


Supplementary data 2. The heat map of Pearson correlation analysis according to the transcriptome samples. NaCl_1， NaCl_2， NaCl_3 and NaCl_4 represented the samples treated with 200 mM NaCl for 0, 2, 4, and 6 days. Con_1, Con_2, Con_3, and Con_4 represented the untreated samples for 0, 2, 4, and 6 days. Screening of differentially expressed genes (DEGs) among samples
