# Supplementary material for: Integrative analysis of morphological, transcriptomic, and metabolomic approaches to uncover the function of flavonoids in the salt stress response of Alhagi camelorum
Source: Front Plant Sci. 2026 Jan 5;16:1678456. doi: 10.3389/fpls.2025.1678456 (PMC12812734; doi:10.3389/fpls.2025.1678456)
Supplement: Supplementary file 3 [file DataSheet3.docx]

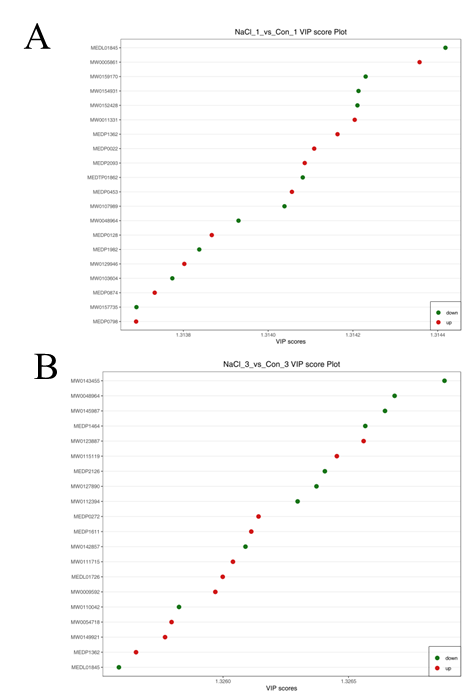


Supplementary data 3 The first twenty up or down DAMs in NaCl_1 vs. Con_1 (A) and NaCl_3 vs. Con_3 (B) groups by VIP score plot analysis.
